# Oxidative stress induced by the Fe^2+^/ascorbic acid system or model ischemia *in vitro*: effect of carvedilol and pyridoindole antioxidant SMe1EC2 in young and adult rat brain tissue

**DOI:** 10.2478/v10102-010-0051-x

**Published:** 2010-12

**Authors:** Zdenka Gáspárová, Olga Ondrejičková, Alena Gajdošíková, Andrej Gajdošík, Vladimír Šnirc, Svorad Štolc

**Affiliations:** 1 Institute of Experimental Pharmacology & Toxicology, Slovak Academy of Sciences, SK-84104 Bratislava, Slovakia; 2 BIONT, Inc., Bratislava, Slovakia

**Keywords:** brain cortex, protein carbonyls, hippocampus, population spike, antioxidants

## Abstract

New effective strategies and new highly effective neuroprotective agents are being searched for the therapy of human stroke and cerebral ischemia. The compound SMe1EC2 is a new derivative of stobadine, with enhanced antioxidant properties compared to the maternal drug. Carvedilol, a non-selective beta-blocker, possesses besides its cardioprotective and vasculoprotective properties also an antioxidant effect. We compared the effect of carvedilol and SMe1EC2, antioxidants with a similar chemical structure, in two experimental models of oxidative stress in young and adult rat brain tissue. SMe1EC2 was found to improve the resistance of hippocampal neurons to ischemia *in vitro* in young and even in 18-month-old rats and inhibited formation of protein carbonyl groups induced by the Fe^2+^/ascorbic acid pro-oxidative system in brain cortex homogenates of young rats. Carvedilol exerted a protective effect only in the hippocampus of 2-month-old rats and that at the concentration 10-times higher than did SMe1EC2. The inhibitory effect of carvedilol on protein carbonyl formation induced by the pro-oxidative system was not proved in the cortex of either young or adult rats. An increased baseline level of the content of protein carbonyl groups in the adult versus young rat brain cortex confirmed age-related changes in neuronal tissue and may be due to increased production of reactive oxygen species and low antioxidant defense mechanisms in the adult rat brain. The results revealed the new pyridoindole SMe1EC2 to be more effective than carvedilol in neuroprotection of rat brain tissue in both experimental models involving oxidative stress.

## Introduction

Major interest is currently focused on the development of new effective strategies and new highly effective agents for the pharmacological therapy of human stroke and cerebral ischemia.

The new substance 2-ethoxycarbonyl-8-methoxy-2,3,4,4a,5,9b-hexahydro-1*H*-pyrido-[4,3b]indolinium chloride with the code SMe1EC2 (m.w. 312.79 Da, chemical purity < 99%), a derivative of stobadine (STO), (both synthetized in the Institute of Experimental Pharmacology and Toxicology, Slovak Academy of Sciences, Slovak Republic) was recently found to have a higher antioxidant capability than the parent compound STO with established neuroprotective and cardioprotective properties (Horáková & Štolc, 1998, Štolc *et al*., 2006). A toxicological and teratological study of SMe1EC2 showed its low toxicity and no embryotoxic and teratogenic effects on developing rats (Štolc *et al*., 2008, Ujházy *et al*., 2008).

Carvedilol, a non-selective beta-blocker with alpha-blocker properties, currently used to treat hypertension, heart failure and coronary artery diseases, has besides its cardioprotective and vasculoprotective properties also antioxidant effects. The antioxidant properties of carvedilol, and its relation to mitochondrial oxidative phosphorylation, calcium homeostasis and energy production, make this drug a unique beta-blocker, reinforcing its advantageous use in cardiac pathologies associated with enhanced cellular oxidative stress. Carvedilol administered subcutaneously directly after transient forebrain ischemia protected a population of neurons in the hippocampal CA1 area in gerbils (Strosznajder *et al*., 2005). Thus carvedilol raises high expectations also in the therapy of ischemia.

**Figure 1:**
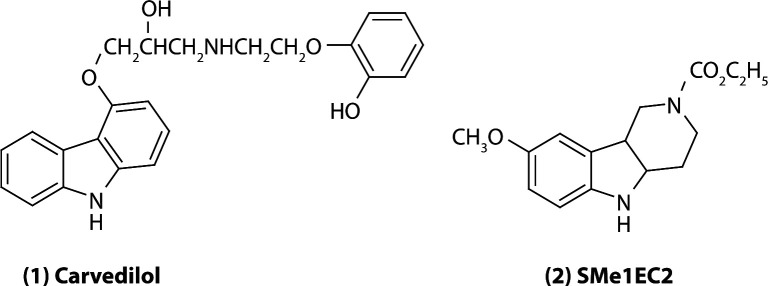
Chemical formula of (1) carvedilol and (2) SMe1EC2.

Both compounds tested, carvedilol and SMe1EC2, have a tri-cyclic basal skeleton bridged by the NH group. This represents the active site of the molecule responsible for interaction with free radicals (Figure 1). The antioxidant activity of both molecules is caused by the ability of the NH group to scavenge radicals by abstraction of the hydrogen from the NH group and by subsequent formation of a more stable N radical. Both compounds have an electron-donor group on the aromatic skeleton, which increases the stability of the N radical and thus its viability.

In two experimental models involving oxidative stress on rat brain tissues, we studied the effect of the new pyridoindole SMe1EC2, derived from the neuroprotective and cardioprotective drug STO, and of carvedilol, a beta adrenoreceptor antagonist with potent antioxidant properties. We focused on 1) comparison of the effect of carvedilol and SMe1EC2 on the resistance of the rat hippocampus exposed to model ischemia *in vitro* (transient glucose/oxygen deprivation followed by reoxygenation), and on 2) comparison of their effect on protein carbonyl formation induced by the Fe^2+^/ascorbic acid pro-oxidative system in the rat brain cortex, both in young and adult rats.

## Methods

### Animals

Male Wistar rats aged 2, 10 and 18 months (weight 216 ± 8 g; 450 ± 8 g and 497 ± 7 g, respectively) (n=20, n=22 and n=15, respectively) from the breeding station Dobrá Voda (Slovak Republic, reg. No. SK CH 4004) were used in electrophysiological and biochemical experiments. The rats had free access to water and food pellets and were kept on a 12/12 h light/dark cycle. All procedures involving animals were performed in compliance with the Principles of Laboratory Animal Care issued by the Ethical Committee of the Institute of Experimental Pharmacology and Toxicology, Slovak Academy of Sciences and by the State Veterinary and Food Administration of the Slovak Republic.

### Drugs

The pyridoindole derivative SMe1EC2 was synthetized in the Institute of Experimental Pharmacology and Toxicology, Slovak Academy of Sciences, Slovak Republic.

Carvedilol, (±)-1-(Carbazol-4-yloxy)-3-[[2-(o-methoxyphenoxy)ethyl]amino]-2-propanol was obtained from La Roche (Mannheim, Germany). Stock solution of carvedilol in the concentration of 1 mmol/l was prepared by dissolution in wine acid and distilled water, heated up to 37 °C and sonicated three times for 5 min.

### Oxygen/glucose deprivation and field action potential in rat hippocampal slices

Rat hippocampal slices (400 µm) were prepared by a conventional technique described in detail earlier (Vlkolinský & Štolc, 1999, Gáspárová *et al*., 2006). Bipolar wire electrodes were used to stimulate Schäffer collaterals evoking trans-synaptically activity in the CA1 area. Field action potential (FAP) was registered in the CA1 pyramidal cell layer by a glass microelectrode and stored in the computer for further analysis. Oxygen/glucose deprivation was obtained by replacement of the gas mixture containing O_2_ with the gas mixture with N_2_ by switching the valves and by replacement of the artificial cerebrospinal fluid (ACSF) equilibrated with oxygen to oxygen-free ACSF, with glucose diminished from 10 to 4 mmol/l. Oxygen/glucose deprivation elicited a decrease of FAP with its subsequent decay. Hippocampal slices were exposed to transient 6-min hypoxia/hypoglycemia followed by 20-min reoxygenation. Recovery of FAP after hypoxia/hypoglycemia was monitored during the 20-min reoxygenation, while population spike (PS) amplitude was measured in a later analysis. Each drug tested was present in the superfusing ACSF throughout the whole experiment: 30 min before oxygen/glucose deprivation, during 6-min hypoxia/hypoglycemia and during 20-min reoxygenation.

### Pro-oxidative system of Fe^2+^/ascorbic acid and protein carbonyl formation in rat brain cortex

Protein carbonyl formation was determined by the method of Levine and coworkers (1990), modified by Blackburn and coworkers (1999) where 2,4-dinitrophenylhydrazine reacts with the protein carbonyl group and protein hydrazon is generated, which is detected spectrophotometrically with absorbance maximum at 360–370 nm. Proteins were detected spectrophotometrically in the same sample at 280 nm. Homogenate of the rat brain cortex (10%) was used. The pro-oxidative system was comprised of FeSO_4_ (0.1 mmol/l) and ascorbic acid (0.5 mMmol/l).

## Results

### Effect of SMe1EC2 and carvedilol during model ischemia *in vitro*

**Figure 2:**
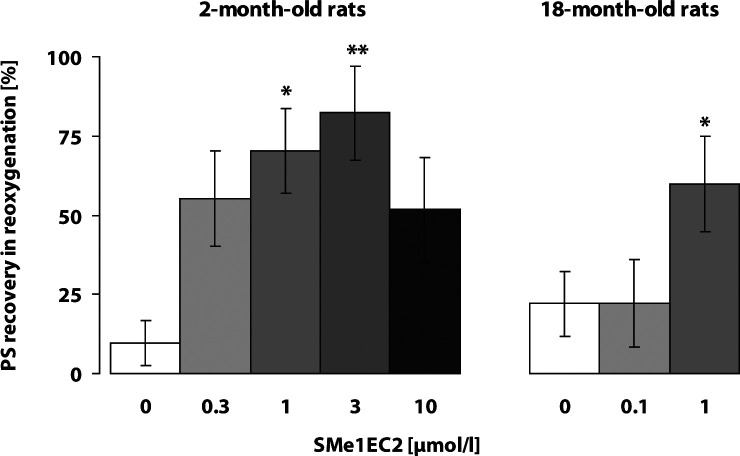
Effect of SMe1EC2 on resistance of the CA1 neurons in hippocampus exposed to transient ischemia *in vitro*: 6-min hypoxia with hypoglycemia followed by 20-min reoxygenation. Ten to 12 slices from 6–8 rats were used in each experimental group. Cessation of field action potential and PS amplitude recovery were monitored. Significant difference in the PS amplitude recovery between untreated and treated slices at the end of 20-min reoxygenation was calculated by Student' t-test,*p<0.05, **p<0.01.

**Figure 3:**
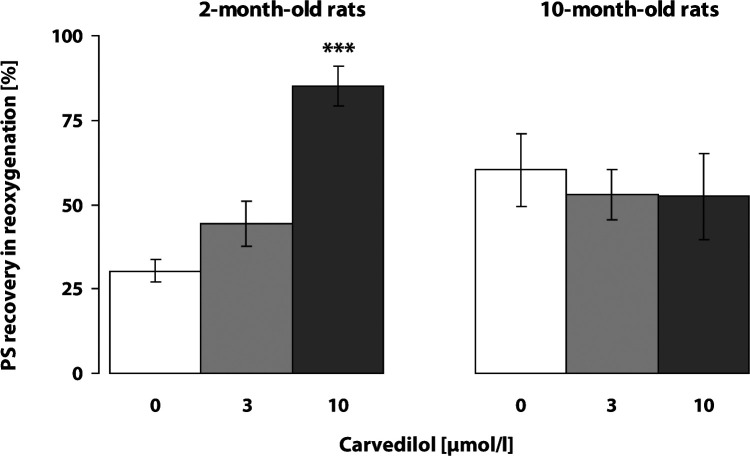
Effect of carvedilol on resistance of CA1 neurons in hippocampus exposed to transient ischemia *in vitro*: 6-min hypoxia with hypoglycemia followed by 20-min reoxygenation. Nine to 11 slices from 6–8 rats were used in each experimental group. Cessation of field action potential and PS amplitude recovery were monitored. Significant difference in the PS amplitude recovery between untreated and treated slices at the end of 20-min reoxygenation was calculated by Student' t-test, ***p<0.001.

Transient 6-min ischemia *in vitro* (hypoxia with hypoglycemia) elicited a decrease and failure of electrically evoked response recorded in the pyramidal neurons of the CA1 area in the rat hippocampus. Untreated control slices showed low recovery of electrically evoked responses at the end of 20-min reoxygenation. SMe1EC2 (1 and 3 µmol/l) significantly improved recovery of the PS magnitude at the end of reoxygenation in the hippocampus of the young 2-month-old rats and established a neuroprotective effect even in the18-month-old rats (1 µmol/l) (Figure 2). Carvedilol, in the concentration of 10 µmol/l, significantly improved the resistance of hippocampal CA1 neurons to transient ischemia *in vitro,* yet only in young rats, while in 10-month-old rats it had no protective effect at any concentration tested (3 and 10 µmol/l) (Figure 3).

### Effect of SMe1EC2 and carvedilol in Fe^2+^/ascorbic acid system

**Figure 4:**
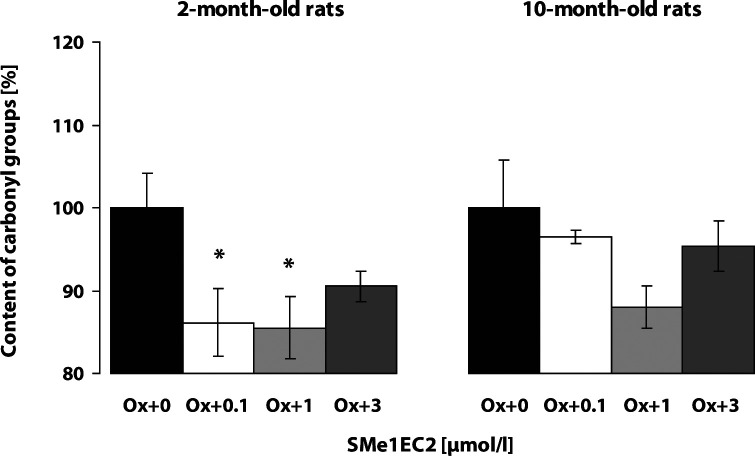
Effect of SMe1EC2 on Fe^2+^/ascorbic acid induced protein carbonyl formation in brain cortex. Rat brain cortex homogenates (n=5–6 samples in each group) from 5–6 rats were used. The content of protein carbonyl groups was determined spectrophotometrically. The content of carbonyls in the presence of the pro-oxidative system without the drug tested was considered 100% (Ox+0). Inhibitory effect of the antioxidant tested was monitored (Ox+0.1; Ox+1; Ox+3 µmol/l of SMe1EC2). Significant difference in protein carbonyl content between the untreated oxidation modified brain cortex homogenates and the treated oxidation modified homogenates was calculated by Student' t-test, *p<0.05.

**Figure 5:**
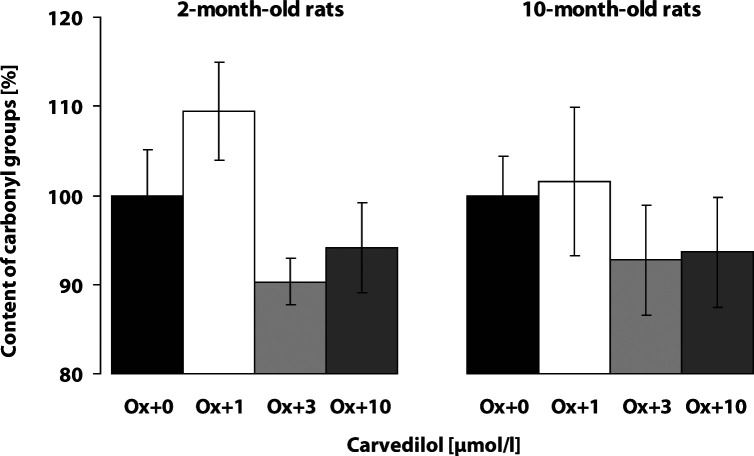
Effect of carvedilol on Fe^2+^/ascorbic acid induced protein carbonyl formation in brain cortex. Rat brain cortex homogenates (n=5–6 samples in each group) from 5–6 rats were used. The content of protein carbonyl groups was determined spectrophotometrically. The content of carbonyls in the presence of the pro-oxidative system without the drug tested was considered 100% (Ox+0). Effect of the drug tested on protein carbonyl content was monitored (Ox+1; Ox+3; Ox+10 µmol/l of carvedilol).

The baseline level of protein carbonyl groups in the cortex of control rats was significantly higher in the adult 10-month-old rats (2.12 ± 0.14 nmol/mg prot., n=20) as compared to the young 2-month-old rats (1.72 ± 0.12 nmol/mg prot., n=22; *p*<0.05). The Fe^2+^/ascorbic acid system induced marked oxidative modification of proteins in both the young and adult rat cortex homogenates, resulting in an increase of the content of protein carbonyl groups (*p*<0.001). The increase of protein carbonyl formation was of similar intensity in the young rat cortex as in the adult one (187.76 ± 6.20%; n=14 and 178.33 ± 8.24%; n=14, respectively). The compound SMe1EC2 protected brain cortex tissue against the oxidative damage induced by the Fe^2+^/ascorbic acid pro-oxidative system in 2-month-old rats even in a very low concentration (0.1 µmol/l) and showed the best effect in the concentration of 1 µmol/l (Figure 4). Carvedilol did not significantly suppress the Fe^2+^/ascorbic acid system induced carbonyl formation in the cortex of either young or adult rats (Figure 5).

## Discussion

To date many natural and synthetic compounds have been established possessing antioxidant properties. Antioxidants and radical scavengers may protect the nervous system against toxic effects of increased levels of reactive oxygen species and free oxygen radicals and thus attract many researchers to study their effect concerning brain protection.

Carvedilol is a multiple-action antihypertensive agent with a potential for cardiovascular protection beyond the normalization of high blood pressure. It has alpha_1_- and beta-adrenergic receptor blocking action, calcium channel blocking action, suppressive effect on cardiac necrosis and neuroprotective activities in animal models of brain ischemia and infarction (Ruffolo *et al*., 1990, Rabasseda 1998, Strosznajder *et al*., 2005). Carvedilol exerts an additional neuroprotective activity as a Na^+^ channel modulator and glutamate release inhibitor (Lysko *et al*., 1994). Recently, carvedilol was found to inhibit mitochondrial permeability transition, mitochondrial swelling, oxidation of thiol groups, and to protect mitochondria against oxidative damage induced by the xanthine oxidase/hypoxanthine pro-oxidant system (Oliviera *et al*., 2004, Oliviera *et al*. 2005, Carreira *et al*., 2006). Chronic administration of carvedilol resulted in an improvement of memory retention (evaluated in the Morris water maze task paradigms) and in attenuation of oxidative damage in the streptozotocin induced model of dementia in rats (Prakash & Kumar, 2009). Carvedilol may have a potential in the treatment of neurodegenerative diseases.

The idea of comparing the effect of these two compounds tested was based on their similar chemical structure and their antioxidant properties, proved in several different approaches. At concentrations above 1 µmol/l, carvedilol was found to be a calcium channel antagonist (Ruffolo *et al*., 1990). We tested it in the concentrations of 1, 3 and 10 µmol/l. In our experiments, the reported antioxidant and neuroprotective action of carvedilol was proved only in the hippocampal slices of young 2-month-old rats exposed to transient hypoxia with hypoglycemia followed by reoxygenation.

The compound SMe1EC2, with an antioxidant and neuroprotective effect established previously (Štolc *et al*., 2006; Štolc *et al*., 2008; Gáspárová *et al*., 2009; 2010), improved the resistance of hippocampal neurons exposed to transient ischemia *in vitro* in young and even in 18-month-old rats and significantly reduced the formation of protein carbonyl groups induced by the pro-oxidative system in young rat cortex homogenates. However, no inhibitory effect of SMe1EC2 on protein carbonyl formation was found in adult 10-month-old rats. This finding might be supported by results where the protective effect of some antioxidants, e.g. vitamin E (Sumien *et al*., 2004; Sung *et al*., 2004), melatonin (von Gall & Weaver, 2008), garlic extract (Brunetti *et al*., 2009), and beta-blockers (Gleibus and Lippa, 2007) was not proved either in adult and aged experimental animals or in elderly people. The increased native baseline level of the content of protein carbonyls in adult rats and a consequent further increase of carbonyls due to exposure to a pro-oxidative system, may be the reason for the failed protective effect of each antioxidant tested in adult 10-month-old rats. The increased baseline level of the content of protein carbonyl groups in the brain cortex of 10-month-old versus 2-month-old rats found in our experiments confirmed age-related changes in neuronal tissue. Our results are in good agreement with findings, for example of Balu and co-workers (2005), reporting age-associated increase in protein oxidation and reactive oxygen species production in the cerebral cortex and hippocampus of aged rats. The determined higher level of oxidatively modified proteins in native rat cortex homogenates of 10-month-old rats compared to young ones could be due to a lower baseline level of antioxidants, which was reported in the neuronal antioxidant system of adult and aged animals compared to young ones (Desole *et al*., 1993; Squier, 2001). The assumption has been voiced that oxidative stress is an early event of chronic brain diseases and antioxidant therapy may be beneficial only if given at this stage of the disease process (Sung *et al*., 2004).

Fe (II) is a potential pro-oxidant and can induce cellular oxidative stress. Ascorbic acid is a powerful physiological antioxidant and, in the presence of free Fe (II), it can exhibit pro-oxidant effects *in vitro*. We found that exposure of brain cortex homogenates to this pro-oxidative system induced an increase in protein carbonyl formation of a range comparable in young and adult rats. Thus in the pro-oxidative system tested, no age-dependent difference was found as to the vulnerability of brain tissue to oxidative stress. To date the relation between oxidant status and antioxidant defense mechanisms in aged animals of different species, organs or sexes has been investigated extensively, yet the results found have frequently been contradictory. Further studies are needed to elucidate these mechanisms and to determine the main signal pathways responsible for changes associated with aging.

## Conclusion

The compound SMe1EC2 showed a significant neuroprotective effect in both experimental models involving oxidative stress and it was effective in lower concentrations than carvedilol. On the basis of previous results obtained with SMe1EC2, along with the findings reported here, we suggest that the new pyridoindole derivative SMe1EC2 is a new prospective and promising compound which might find a beneficial use in the treatment of neuronal impairment, such as stroke and brain ischemia.
